# Assessing the Impact of Telemedicine on Patient Satisfaction Before and During the COVID-19 Pandemic

**DOI:** 10.3390/healthcare13172095

**Published:** 2025-08-23

**Authors:** Ashiat Adeogun, Misa Faezipour

**Affiliations:** 1 Healthcare Informatics Program, College of Basic and Applied Sciences, Middle Tennessee State University, Murfreesboro, TN 37132, USA; aaa2ev@mtmail.mtsu.edu; 2 Department of Engineering Technology, Middle Tennessee State University, Murfreesboro, TN 37132, USA

**Keywords:** telemedicine, patient satisfaction, systems thinking, causal model, COVID-19 pandemic, chronic conditions

## Abstract

**Objectives:** The adoption of telemedicine, which is the delivery of healthcare services through digital platforms, exploded during the COVID-19 pandemic as a means to ensure the continuity of care while minimizing infection risks. While this modality improved access and convenience for many, disparities in adoption have emerged, particularly in rural and underserved populations. This study aims to evaluate the impact of telemedicine on patient satisfaction before and during the pandemic, with a focus on chronic disease management—notably hypertension—and to identify factors influencing the equitable adoption of telehealth. **Methods and Procedures:** This study used a mixed method approach, combining quantitative survey data and causal loop modeling to analyze patient satisfaction levels and the interplay between telehealth adoption, healthcare access, and demographic disparities. Patient-reported satisfaction data were collected in two time periods—before and during the pandemic. Causal modeling was used to explore systemic relationships between provider support, technology access, patient engagement, and health equity. **Results:** The findings revealed that telemedicine significantly enhanced healthcare access during the pandemic, with a notable increase in patient satisfaction scores. Patients with chronic conditions, especially those diagnosed with hypertension, reported the improved continuity of care and reduced geographic barriers. However, disparities in telehealth access were more pronounced in non-metropolitan areas and among older adults and minority populations. Causal analysis highlighted key enablers of telehealth success, i.e., provider support, digital literacy, and access to reliable internet and devices. **Conclusions:** Telemedicine presents a transformative solution for equitable healthcare delivery, especially in chronic disease management. Strategic efforts are needed to address adoption disparities and ensure the sustained and inclusive integration of telehealth after the pandemic.

## 1. Introduction

The COVID-19 pandemic dramatically reshaped the global healthcare landscape, prompting a rapid shift toward telemedicine to maintain the continuity of care while minimizing infection risk for patients and providers alike [1]. Telemedicine refers to the delivery of healthcare services through electronic and telecommunication technologies, enabling remote consultations, diagnosis, monitoring, and treatment. Its applications range from virtual patient visits and remote nursing to psychiatric rehabilitation and chronic disease management. By minimizing the need for physical visits, telemedicine helps reduce healthcare costs, travel burdens, and diagnostic delays while enhancing access and convenience [2,3].

Despite these advantages, patient satisfaction, which is a key indicator of healthcare service quality, varies considerably based on the mode of delivery. The sudden expansion of telemedicine during the pandemic revealed critical disparities in access and engagement, especially across different age groups, geographic regions, and chronic condition populations.

As healthcare systems transition toward a hybrid care model that blends in-person and virtual services, it is essential to assess how telemedicine impacts patient satisfaction and equity. Factors such as ease of access, communication quality, and patient outcomes play vital roles in determining whether telemedicine can be a sustainable long-term solution [4,5]. Understanding how these factors shifted during the pandemic can guide the development of more inclusive and effective telehealth systems.

To gain a deeper understanding of the underlying mechanisms shaping patient satisfaction, this study employs a mixed method approach that integrates descriptive and inferential statistical analysis with a conceptual causal loop model. Causal loop modeling, grounded in systems thinking, helps visualize complex feedback relationships among variables such as telehealth adoption, provider support, digital infrastructure, and chronic condition management [6,7,8]. These dynamic interactions offer insights into the structural drivers of satisfaction, disparities in access, and behavioral trends within the telemedicine ecosystem.

A unique contribution of this study lies in the integration of systems thinking through causal loop modeling with a quantitative analysis of nationally representative CDC survey data. This hybrid approach provides a systems-level framework for exploring how digital inequities and patient–provider dynamics shape satisfaction trends across demographic and geographic subgroups. By contextualizing observed patterns within a dynamic feedback structure, this research offers conceptual insights into the forces influencing equitable telemedicine delivery.

This study specifically examines changes in patient satisfaction with telemedicine services before and during the COVID-19 pandemic. It explores how access to care, perceptions of healthcare quality, and patient engagement contributed to these changes. The findings identify key drivers of satisfaction across both periods and offer policy and design recommendations to strengthen future telehealth services.

## 2. Related Works

Several studies have examined the adoption and impact of telemedicine during the COVID-19 pandemic, particularly with respect to patient satisfaction, chronic disease management, and healthcare access. Prior work using CDC survey data has largely focused on descriptive statistics to report adoption rates and general trends in satisfaction across demographics [9,10]. These studies have highlighted the digital divide in rural versus urban areas, the role of chronic conditions such as hypertension and diabetes, and the expansion of telehealth services during the pandemic [11,12].

However, few studies have applied a systems thinking approach to interpret these trends. Our study differs by integrating conceptual causal modeling through a Causal Loop Diagram (CLD) with quantitative analysis. This hybrid method provides a more holistic view of the feedback mechanisms shaping patient satisfaction and telehealth equity. Unlike prior research, we compare satisfaction before and during the pandemic, segmented by age, race/ethnicity, and geography, allowing us to uncover structural dynamics that drive disparities in telemedicine use and outcomes.

## 3. Methods

### 3.1. Study Design

This study employed a mixed method design, integrating quantitative analysis with systems thinking through causal loop modeling in order to examine factors influencing patient satisfaction with telemedicine.

### 3.2. Data Source and Sample

The quantitative component utilized a publicly available dataset from the Centers for Disease Control and Prevention (CDC) [9], comprising 804 records categorized by demographic variables and health indicators. Participants were grouped into subpopulations based on characteristics such as age, race, and geographic location, and responses were segmented into pre-pandemic and during-pandemic periods to provide a nationally representative sample.

### 3.3. Survey Instrument

The data utilized in this study were drawn from a nationally representative, publicly available survey administered by the CDC [9]. The instrument included a series of closed-ended questions addressing demographic characteristics (such as, age, race/ethnicity, healthcare access, chronic disease status, and telemedicine utilization).

Patient satisfaction with telemedicine was measured using a single Likert-scale item, in which respondents rated their satisfaction with telehealth services on a five-point scale ranging from 1 (“Very dissatisfied”) to 5 (“Very satisfied”). This item served as the primary outcome variable and is referred to in the analysis as the Patient Satisfaction Response (’P. Response’). Higher values indicate greater levels of satisfaction.

In addition to satisfaction, respondents were asked whether their healthcare provider offered telemedicine services prior to the COVID-19 pandemic. Response options included “Yes,” “No,” “Do not know,” and missing or non-responses. These responses were stratified by demographic subgroups, including age (18–44 years, 45–64 years, and 65 years and older) and race/ethnicity (e.g., White non-Hispanic), allowing for comparative analyses across subpopulations and pandemic phases (pre-pandemic vs. during-pandemic).

Although the instrument was not developed by the authors, it adheres to the CDC’s standardized data collection protocols and employs measures that are consistent with validated tools commonly used in public health research.

### 3.4. Data Analysis

The quantitative analysis primarily employed descriptive statistics and one-sample *t*-tests to explore subgroup differences in patient satisfaction across key demographic variables. This approach was selected to provide an initial assessment of satisfaction patterns using a large, publicly available dataset.

Due to the anonymized and cross-sectional nature of the CDC dataset, individual-level longitudinal data were not accessible. As a result, advanced statistical techniques such as difference-in-differences (DiD), panel data analysis, or multivariate regression could not be applied. While the use of one-sample *t*-tests limits the ability to draw causal inferences or fully control for confounding variables, it aligns with the exploratory intent of this study and supports the integration of conceptual causal modeling through systems thinking.

Although effect sizes (such as Cohen’s d) were not included in the initial analysis, we acknowledge their importance in assessing the practical significance of group differences. We recommend incorporating such measures in future research to enhance the interpretability and robustness of statistical findings.

### 3.5. Causal Model

This study employs a systems thinking approach through the development of a conceptual Causal Loop Diagram (CLD) to illustrate feedback relationships among telemedicine adoption, patient satisfaction, and health equity. The CLD serves as a qualitative modeling tool that integrates insights from the CDC’s telemedicine dataset with system dynamics theory. This approach provides a holistic perspective on the interconnected factors influencing telehealth outcomes, such as technological access, provider support, and chronic disease management, which are not easily captured through traditional statistical methods.

We acknowledge that the CLD developed here is qualitative and does not quantify causal relationships in a statistical sense. Instead, it serves as a conceptual tool to interpret observed patterns in the dataset, such as variations in satisfaction across demographic and geographic subgroups and to propose plausible mechanisms behind those patterns. Unlike purely descriptive studies, our framework visually and theoretically synthesizes the behavioral and structural drivers affecting telehealth equity and engagement [8].

Although we did not employ formal statistical causal inference methods such as structural equation modeling or Granger causality, our Causal Loop Diagram (CLD) was conceptually validated by aligning its feedback loops with empirical patterns identified in our dataset and corroborating them with the prior literature. For example, loops R1 (Telehealth Access and Satisfaction), R2 (Provider Support and Satisfaction), and B1 (Reduced Hospital Readmissions and Chronic Disease Management) were grounded in statistically significant trends in healthcare access, chronic disease outcomes (e.g., higher satisfaction among patients with hypertension compared to those with diabetes or asthma), and demographic disparities (e.g., metropolitan vs. non-metropolitan differences, as well as racial/ethnic subgroup variations). Similarly, loops R3 (Technology Literacy and Engagement) and R4 (Technology Innovation and Adoption) were informed by observed associations between digital literacy, platform usability, and telehealth uptake. This iterative mapping ensured that the CLD not only reflected established systems thinking principles but also accurately represented the relationships observed in our analysis, thereby strengthening its validity as a framework for interpreting telemedicine adoption and equity dynamics.

**Figure 1:**
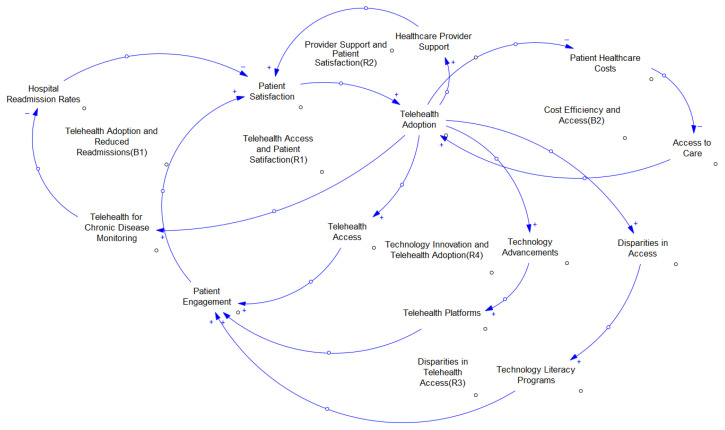
Telemedicine causal model.

Figure 1 illustrates key reinforcing and balancing loops. For example, Reinforcing Loop R1 captures the feedback between telehealth access and satisfaction, while Loop R2 incorporates the influence of provider support. Loop B1 suggests that increased telemedicine adoption may reduce hospital readmissions, improving chronic disease management outcomes. Additional loops (R3 and R4) model the role of technological literacy and digital disparities, particularly in non-metropolitan settings.

This qualitative model, grounded in both survey findings and the prior literature, enables a systems-level understanding of telemedicine delivery dynamics and identifies leverage points for policy interventions aimed at promoting equitable care.

Figure 1 represents the causal model in which several reinforcement and balancing loops are identified. A reinforcing loop is a feedback loop in which a change in one part of the system leads to further changes that amplify the initial effect. In system dynamics models, this loop is often depicted with an arrowed circle, which indicates that each element of the loop enhances or amplifies the next, leading to a continuous growth or decline cycle [13]. A balancing loop (or negative feedback loop) in system dynamics works to regulate the system, counteracting changes to maintain stability or move the system towards a goal or equilibrium. This loop seeks to dampen the initial change, keeping the system within certain bounds or reversing the direction of change [13]. For example, Reinforcing Loop 1 (R1) shows that telehealth access and patient satisfaction are positively correlated, whereby greater access to telehealth can increase patient satisfaction, which, in turn, encourages higher adoption rates. Reinforcing Loop 2 (R2) highlights the role of provider support in enhancing patient satisfaction, further driving telehealth usage. On the other hand, Balancing Loop B1 indicates that the adoption of telehealth can reduce hospital readmission, thus improving the efficiency and management of chronic diseases.

The model also demonstrates that technological advances and patient participation are critical drivers of telehealth adoption, while disparities in access can hinder its widespread use, particularly in rural or underserved communities. By incorporating technology literacy programs and addressing disparities, the system can foster greater telehealth equity, as depicted by the loops labeled R3 and R4. This approach helps to pinpoint leverage points where policy interventions can improve telehealth outcomes and patient satisfaction in various populations [8].

## 4. Descriptive Analysis Results

To effectively present the results, each subsection highlights key findings with a clear focus on the management of relevant demographic, geographic, and chronic conditions, providing concise insights supported by evidence from descriptive and inferential statistical analysis. Each of the categories will be shown along with their results.

### 4.1. Healthcare Providers Offered Telemedicine

**Figure 2:**
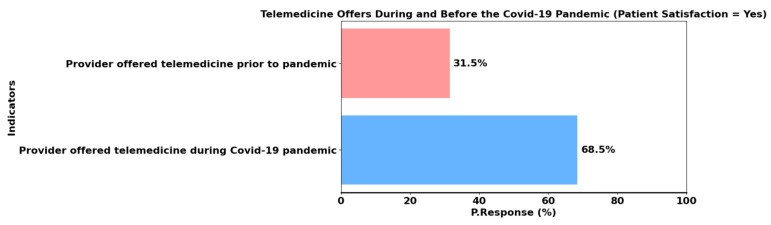
Comparison between telemedicine providers prior and during the pandemic.

We used the Python programming language to perform data analysis. The chart in Figure 2 illustrates the shift in telemedicine offerings before and during the COVID-19 pandemic. Prior to the pandemic, 31.5% of healthcare providers offered telemedicine services. However, during the pandemic, this number increased significantly, with 68.5% of patients adopting telemedicine. This increase highlights the healthcare sector’s rapid response to the challenges posed by the pandemic, as telemedicine became a vital tool for maintaining patient care while minimizing the risk of infection. The data underscore the importance of telemedicine as a flexible and effective solution to ensure access to healthcare during times of crisis, as shown in Figure 2.

### 4.2. Age Group

An age group is a classification of individuals according to their age, often grouped into ranges such as children (0–12 years), adolescents (13–18 years), adults (19–64 years), and older adults or seniors (65 years and older). These categories are commonly used in healthcare to analyze differences in health needs, behaviors, and outcomes across life stages. Age is a significant factor influencing healthcare delivery, as physiological, psychological, and social needs evolve over time [14].

**Figure 3:**
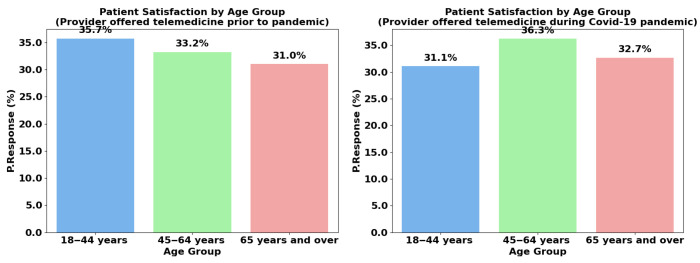
Age group comparison during and prior to the pandemic.

The chart highlights changes in patient satisfaction with telemedicine services across age groups before and during the COVID-19 pandemic, as shown in Figure 3. Before the pandemic, the 18–44-year-old age group had the highest satisfaction (35.7%), followed by the 45–64 years (33.2%) and 65 years and older (31.0%) groups. During the pandemic, satisfaction increased slightly for the 45–64-year-old group (36.3%) and the 65 years of age and older group (32.7%), while satisfaction for the 18–44-year-old group decreased to 31.1%. This shift suggests a change in how different age groups perceived the value of telemedicine during the pandemic.

Middle-aged and older patients benefited the most, likely due to the ability of telemedicine to meet their needs for the management of chronic diseases and the reduced risk of exposure. In contrast, younger patients experienced a drop in satisfaction, possibly because telemedicine did not fully address their healthcare expectations, which may include a preference for in-person interactions. In general, the data indicate that telemedicine was better received by older age groups during the pandemic, highlighting its growing relevance to managing long-term health needs.

### 4.3. Gender

Gender refers to the socially constructed roles, behaviours, expressions, and identities of individuals typically categorized as male, female, or non-binary. In healthcare, gender can influence patient experiences, access to care, and satisfaction with services [15].

**Figure 4:**
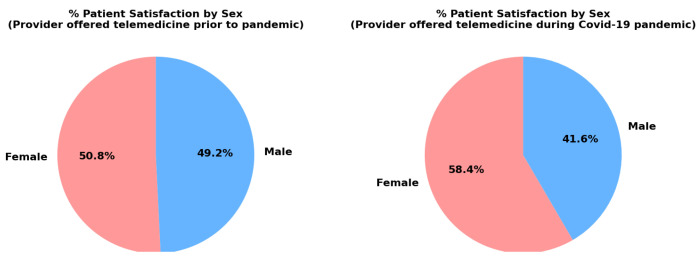
Gender comparison during and prior to the pandemic.

In Figure 4, the charts highlight a shift in telemedicine satisfaction between males and females before and during the pandemic. During the pandemic, a greater proportion of females (58.4%) reported satisfaction with telemedicine compared to males (41.6%). This shift can be attributed to several factors. Firstly, telemedicine offered increased access and convenience, which benefited women who often have caregiving responsibilities. Remote consultations allowed them to save time and overcome transportation barriers [16]. Additionally, women tend to be more proactive in seeking healthcare and managing chronic conditions, making telemedicine an ideal solution to maintain healthcare routines without disrupting their daily responsibilities [17]. The pandemic also heightened safety concerns, and women may have turned to telemedicine to minimize exposure to COVID-19 [18]. Conversely, before the pandemic, men had a higher satisfaction rate with telemedicine (49.2%). This may be explained by men’s greater familiarity with technology, as they are often early adopters of new technologies, and through work-related benefits, as telemedicine allowed men to save time and avoid disruptions to their demanding work schedules [16].

### 4.4. Urbanization

**Figure 5:**
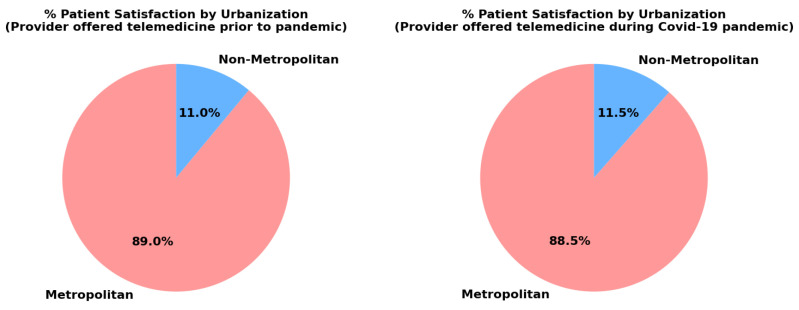
Metropolitan and non-metropolitan differences during and prior to the pandemic.

Urbanization refers to the study of physical locations and environments, such as urban, rural, and suburban areas; influential human behaviors; access to resources; and outcomes. In healthcare, urbanization factors play a critical role in shaping access to care, resource allocation, and health disparities. Urban areas often benefit from concentrated healthcare services and advanced technologies, while rural areas may face challenges such as limited provider availability, longer travel distances, and fewer speciality services [19]. The charts illustrate the difference in patient satisfaction with telemedicine adoption between metropolitan and non-metropolitan areas, both during and before the pandemic, as shown in Figure 5. Metropolitan areas consistently show higher satisfaction rates, which can be attributed to several factors. Firstly, there is better infrastructure in cities, including reliable high-speed internet and advanced healthcare technology, which allows for seamless telemedicine interactions. In contrast, rural areas often face challenges with connectivity, leading to poor video and audio quality during consultations, thus discouraging its use [20]. Secondly, urban healthcare systems tend to adopt new technologies earlier due to having a greater access to resources and skilled professionals. This enables metropolitan patients to benefit from telemedicine services earlier and more frequently than their non-metropolitan counterparts.

Moreover, metropolitan areas have more healthcare providers, offering a wider range of telemedicine services, which leads to easier access to care and reduced wait times. The higher population density in cities may also push providers to adopt telemedicine in order to efficiently manage larger patient volumes. Another important factor is the familiarity with technology in urban populations, who are generally more tech-savvy, making the transition to telemedicine smoother. Conversely, non-metropolitan areas face a digital divide, where patients may lack access to technology or the skills to navigate telemedicine platforms effectively [21].

Rural areas in the United States face significant challenges, including limited access to healthcare resources compared to urban communities. For instance, more than 4.7 million people reside in 460 rural counties across the country that lack general medical or surgical hospital beds. Additionally, 16.4 million rural inhabitants live in areas without access to medical or surgical intensive care unit (ICU) beds [21]. Rural residents tend to have shorter life expectancies than their urban counterparts, and rural households report lower median incomes [22]. In non-metropolitan areas, barriers such as limited internet access and technological literacy significantly hinder the widespread adoption of telemedicine. Many rural patients, especially older adults, may lack familiarity with the necessary devices and software required for virtual consultations. Additionally, rural patients may prefer in-person consultations, viewing them as more effective and trustworthy due to longstanding relationships with local healthcare providers. This perception of telemedicine as being less effective than face-to-face care further contributes to the lower adoption rates in non-metropolitan areas [23].

### 4.5. Race/Hispanic Origin

White non-hispanics refers to individuals who identify as White and do not have Hispanic or Latino origins. Historically, White non-Hispanic individuals in the U.S. have benefited from greater access to healthcare resources, including insurance coverage, advanced technologies, and high-quality providers. However, disparities still exist within this group based on socioeconomic status, rural versus urban living, and education levels [24].

Black non-Hispanic individuals face significant systemic barriers in accessing and receiving equitable healthcare. Historical and ongoing racial discrimination in healthcare systems has led to disparities in treatment outcomes, quality of care, and trust in providers. Studies show that Black patients are less likely to access advanced healthcare services like telemedicine due to socioeconomic disparities, lack of internet access, and provider biases. They often report lower satisfaction levels, which can stem from a lack of cultural competency among providers and systemic inequities in healthcare delivery [25].

The Hispanic or Latino population includes individuals of Spanish-speaking origin, typically from Latin America or Spain. This group often experiences unique challenges in healthcare, such as language barriers, lack of insurance, and limited cultural competency among healthcare providers [26].

The disparities in telemedicine satisfaction rates by race, as shown in the charts, reflect broader systemic inequities in healthcare access and digital literacy. The sharp drop in satisfaction among White non-Hispanic patients may partly be attributed to higher expectations of telehealth systems and better prior access to in-person care, which they may have preferred over virtual consultations. As telemedicine became the default option during the pandemic, many White non-Hispanic patients, particularly in rural areas, faced challenges with healthcare system overloads and inadequate technological infrastructure, leading to dissatisfaction [27].

**Figure 6:**
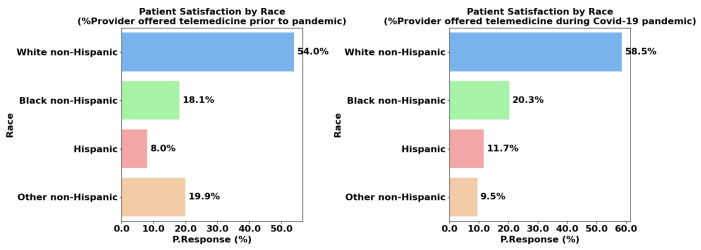
Race disparities during and prior to the pandemic.

In contrast, the increase in satisfaction rates among Black non-Hispanic and Hispanic populations during the pandemic suggests that telemedicine may have mitigated some existing barriers to healthcare for these groups, as shown in Figure 6. Hispanic patients are less likely to engage with telemedicine due to limited English proficiency, lower digital literacy, and affordability issues. Many report dissatisfaction with healthcare interactions due to a lack of language support and culturally appropriate care. Despite these challenges, this group has shown increased telemedicine usage during the pandemic, although satisfaction remains low.

Telemedicine allowed for more accessible care in rural communities, where in-person healthcare options were historically limited. This improvement is particularly notable in urban areas where telemedicine platforms were more robust, offering a viable alternative for populations with lower access to quality healthcare [5]. Studies have also highlighted how the pandemic amplified efforts to reduce healthcare disparities through initiatives like telehealth outreach, leading to improved satisfaction among minority groups, who previously faced significant barriers to care due to cost, transportation, and racial discrimination [28].

However, disparities in technology literacy, language barriers, and internet access still disproportionately affect minority populations, particularly in rural settings, contributing to continued disparities in telemedicine adoption and satisfaction. Addressing these gaps will require ongoing investment in telemedicine infrastructure, culturally sensitive care models, and digital literacy programs, especially for non-metropolitan regions and minority communities. Ensuring equitable access to telemedicine will play a critical role in reducing healthcare inequities and improving overall patient satisfaction moving forward [29].

### 4.6. Chronic Conditions Management

Hypertension, diabetes, and asthma are chronic conditions that necessitate regular monitoring and proactive management to prevent complications. Hypertension, characterized by persistently elevated blood pressure, is a leading risk factor for cardiovascular diseases. Patients with hypertension often require frequent blood pressure monitoring, medication adjustments, and lifestyle interventions such as diet and exercise changes. These management strategies can be implemented effectively through telemedicine, which offers convenient remote monitoring, patient education, and virtual consultations without the need for frequent in-person visits [30].

Diabetes is a complex metabolic disorder that involves impaired insulin production or function, resulting in elevated blood glucose levels. Managing diabetes requires constant attention to glucose control, insulin administration, dietary regulation, and physical activity. Telemedicine poses challenges for diabetes management, as it lacks the ability to provide physical examinations and immediate intervention for acute complications like diabetic ketoacidosis or severe hypoglycemia. However, for routine monitoring, telemedicine can offer virtual consultations, remote glucose monitoring, and education, though its limitations may explain the lower satisfaction rates [31].

Asthma is a chronic inflammatory condition of the airways that causes recurrent episodes of wheezing, shortness of breath, chest tightness, and coughing. While telemedicine can support asthma management by providing education, symptom monitoring, and medication adjustments, severe asthma often requires physical evaluations such as lung function tests (spirometry) or immediate intervention for exacerbations. This need for hands-on management limits the effectiveness of telemedicine for patients with moderate to severe asthma [32].

**Figure 7:**
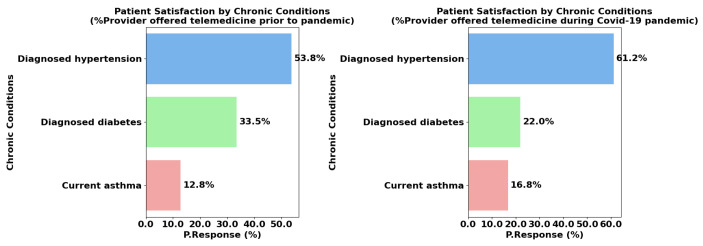
Chronic conditions management during and prior to the pandemic.

In Figure 7, the comparison of patient satisfaction according to chronic conditions provides key insights into how patients with these conditions responded to telemedicine services during and prior to the COVID-19 pandemic. For patients with diagnosed hypertension, satisfaction levels were consistently high, with 62.8% reporting satisfaction during the pandemic and 56.7% before the pandemic. This suggests that telemedicine was particularly beneficial for hypertension patients, who could effectively monitor and manage their condition through remote care, avoiding frequent clinic visits and maintaining control over their health.

In contrast, satisfaction levels for patients with diagnosed diabetes and asthma were lower. During the pandemic, 19.1% of diabetes patients and 18.1% of asthma patients expressed satisfaction with telemedicine. Before the pandemic, satisfaction rates were slightly higher, with 19.6% of diabetes patients and 23.7% of asthma patients reporting positive experiences. The lower satisfaction for these conditions may reflect the complexity of their management, which often requires more hands-on care. For diabetes, in-person care can be crucial for assessing complications, adjusting treatment, and providing immediate support in emergencies, which telemedicine may not fully address [32]. Similarly, asthma patients may have found it challenging to manage their condition effectively without in-person assessments, especially during the pandemic when access to healthcare resources may have been limited.

The slight decline in satisfaction among asthma patients during the pandemic (from 23.7% to 18.1%) could be due to the strained healthcare system, which affected the quality and availability of telemedicine services. These patterns highlight the varying effectiveness of telemedicine across different chronic conditions, with hypertension management showing the highest patient satisfaction, while more complex conditions like diabetes and asthma may require more comprehensive in-person care to meet patient needs.

### 4.7. Inferential Statistical Analysis

The goal for inferential analysis is to assess whether significant differences exist between various demographic groups and telemedicine satisfaction, both before and during the pandemic. This analysis helps identify patterns and key factors that may influence patient satisfaction and the adoption of telemedicine services. The following describe the confident interval and *p*-value equations.

#### 4.7.1. The 95% Confidence Interval (CI)

The confidence interval formula is given as follows:

$$CI=\bar{x}\pm \left(t_{\alpha /2}\cdot \frac{s}{\sqrt{n}}\right)$$

where

- $\bar{x}$ = sample mean;
- $t_{\alpha /2}$ = critical value from the t-distribution for 95% confidence (based on degrees of freedom, n−1);
- *s* = sample standard deviation;
- *n* = number of data points (sample size).

The confidence interval range is as follows:

$$\left(\bar{x}-t_{\alpha /2}\cdot \frac{s}{\sqrt{n}},\bar{x}+t_{\alpha /2}\cdot \frac{s}{\sqrt{n}}\right)$$

#### 4.7.2. *p*-Value (One-Sample *t*-Test)

The formula for the t-statistic in a one-sample *t*-test is as follows:

$$t=\frac{\bar{x}-\mu_{0}}{\frac{s}{\sqrt{n}}}$$

where

- *t* = t-statistic;
- $\bar{x}$ = sample mean;
- $\mu_{0}$ = hypothesized population mean (usually 0 for a one-sample *t*-test);
- *s* = sample standard deviation;
- *n* = number of data points (sample size).

The P. Response dataset contains 102 observations, with a mean value of approximately 479, indicating the average response across the dataset. The variability in responses is significant, as shown by the standard deviation of 431. The minimum recorded response is 20, and 25% of the data points fall at or below 125. The median, or the 50th percentile, is 350, meaning that half of the responses are above this value and half are below. At the 75th percentile, the responses are observed at 700 or lower. The maximum response recorded is quite high (at 2200), showing the presence of some extreme values in the dataset. This suggests a wide range of responses, with many values being concentrated in the lower end, along with some very high outliers. The provided Table 1 presents the results of a descriptive and inferential statistical analysis using *t*-tests, examining the differences in patient responses across various demographic and health-related subgroups. The analysis evaluates the influence of factors such as sex, age, education, urbanization, race/Hispanic origin, and chronic conditions on mean patient responses. Confidence intervals (95% CIs) are included to indicate the range in which the true mean likely falls, and *p*-values are presented to determine the statistical significance of the differences.

The analysis of patient satisfaction responses (P. Response) across age subgroups reveals significant findings. For the 18–44 year-old group, the mean P. Response is 434.33, (95% CI: 157.73, 710.94) and a *p*-value of 0.0100, indicating statistical significance. The 45–64 year-old group shows a slightly higher mean P. Response of 475.00, (95% CI: 107.68, 842.32) and a *p*-value of 0.0209, which is also statistically significant. Similarly, the 65 years and over group has a mean P. Response of 432.17, (95% CI: 124.43, 739.91) and a *p*-value of 0.0154, confirming significance. The findings suggest that all age subgroups exhibit meaningful levels of patient satisfaction, as evidenced by statistically significant *p*-values across the groups. The overlapping confidence intervals indicate some similarity in the range of satisfaction levels, although specific differences in the mean responses may warrant further investigation to explore potential variations between subgroups.

In terms of sex, the mean patient response for males is 606 (95% CI: 271.80–941.87), while females have a slightly higher mean response of 775 (95% CI: 218.99–1331.01). The *p*-values for both sexes are statistically significant, with *p*-values of 0.006 for males and 0.016 for females. The wide confidence intervals for both groups suggest variability in satisfaction or adoption levels, but generally, females might have a slightly higher perception or engagement with telemedicine than males. This insight could guide telemedicine strategies that consider gender-specific needs and expectations.

In terms of urbanization, respondents from metropolitan areas show a markedly higher mean patient response of 1146 (95% CI: 320.17, 1971.83) compared to non-metropolitan respondents, who have a mean response of 146 (95% CI: 31.77, 261.56). The *p*-values for both groups are significant, with 0.016 for metropolitan and 0.022 for non-metropolitan. This significant difference may indicate a higher adoption or satisfaction with telemedicine in metropolitan areas, likely due to better internet infrastructure, more tech-savvy populations, and easier access to digital health resources. Conversely, the low mean response in non-metropolitan areas highlights the need for targeted efforts to improve telemedicine access and support in rural communities.

For race/Hispanic origin, White Non-Hispanic individuals have a higher mean response, at 948 (95% CI: 372.52–1524.82), while Black Non-Hispanic report a value of 325 (95% CI: 93.29–556.71), Hispanic report a value of 172 (95% CI: 34.55–310.79), and Other Hispanic report a value of 220 (95% CI: 4.92–436.75); these groups show lower mean responses with narrower confidence intervals, respectively. The *p*-values for all racial subgroups are statistically significant, with *p*-values of 0.0129, 0.0029, 0.0082, and 0.0093, indicating that race or Hispanic origin significantly influences patient responses. This difference may indicate disparities in telemedicine adoption across racial and ethnic groups, possibly due to factors like access to technology, cultural preferences, or trust in digital healthcare. Lower responses among minority groups suggest that telemedicine programs could benefit from culturally tailored outreach and support to increase adoption and engagement.

Lastly, for chronic conditions, individuals with diagnosed hypertension have a higher mean response, at 601 (95% CI: 189.52–1012.48) compared to those with diabetes (263 (95% CI: 76.23–450.44)) and asthma (158 (95% CI: 39.99–276.68)). The *p*-values for all chronic conditions are significant, with 0.013 for hypertension, 0.015 for diabetes, and 0.018 for asthma, indicating that different chronic conditions impact patient responses. These findings suggest that patients with hypertension might be more receptive to or have higher satisfaction with telemedicine services, perhaps due to the feasibility of managing hypertension through virtual consultations. In contrast, patients with diabetes and asthma may require more in-person care or specialized interventions, which could impact their engagement with telemedicine. Understanding these preferences allows providers to better tailor telemedicine options for chronic disease management.

Although the *p*-values in Table 1 indicate statistically significant differences across demographic groups, the wide confidence intervals, particularly for the metropolitan and race subgroups, suggest considerable variability in responses. This may reflect sample size limitations or heterogeneity within subgroups. For example, the satisfaction scores among metropolitan respondents show a higher mean but also a wide 95% CI (320.17–1971.83), indicating inconsistent experiences that warrant further investigation.

Overall, the significant *p*-values (all less than 0.05) across all subgroups suggest that factors such as sex, age, education, urbanization, race, and chronic conditions have a meaningful influence on patient responses. The differences observed across these variables are unlikely to be due to chance, underscoring their importance in understanding patient satisfaction and behavior.

## 5. Discussion

This paper contributes to the existing literature by providing a comprehensive analysis of the impact of telemedicine on patient satisfaction during the COVID-19 pandemic, specifically focusing on changes in satisfaction levels and the factors influencing these outcomes in pre- and during-pandemic contexts.

While the CLD in this study is not statistically validated, it offers a valuable theoretical lens for interpreting the complex interplay between demographic trends and systemic healthcare barriers. For instance, feedback loops around technological literacy and chronic disease management help explain the observed variation in patient satisfaction across age and geographic subgroups. This approach provides groundwork for future research to build statistically calibrated system dynamics or agent-based models using richer longitudinal datasets.

We acknowledge the limitations of using basic statistical techniques such as one-sample *t*-tests, particularly in light of the wide confidence intervals and the absence of effect size reporting. These limitations stem from the structure of the available dataset, which lacked panel or repeated measures data. Future studies should consider applying multivariate regression models or difference-in-differences designs with richer longitudinal datasets to validate the observed patterns and better assess causal relationships.

Telemedicine has benefits and limitations, and there are strategies to help enhance telemedicine adoption. There are many benefits including the following:

*Improved Access to Healthcare*: Telemedicine significantly increases access to healthcare, particularly for populations in metropolitan areas where digital infrastructure is more advanced. In these areas, healthcare providers can manage larger patient volumes more efficiently, allowing patients to access care more quickly. During the pandemic, telemedicine became a vital resource even in non-metropolitan areas, although disparities persisted due to connectivity issues and limited access to technology in rural regions. This digital divide highlights the need for enhanced infrastructure in underserved communities to fully realize the potential of telemedicine [11].

*Enhanced Patient Satisfaction:* The introduction and expansion of telemedicine have led to improved patient satisfaction, particularly among individuals with chronic conditions like hypertension and diabetes. These patients benefit from regular, flexible healthcare check-ins without the need for in-person visits. Notably, specific demographic groups, such as White non-Hispanic populations, reported high satisfaction levels with telemedicine, although there was a slight dip in satisfaction during the pandemic, likely due to the strain on healthcare systems during this period [10]. Nonetheless, telemedicine has proven to be a valuable tool in increasing patient engagement and improving overall satisfaction with healthcare services.

*Convennice and Flexibility:* One of the most appreciated benefits of telemedicine is the convenience it offers. Patients can schedule consultations and receive medical care remotely, eliminating the need for travel, which can be time-consuming and costly. This benefit is particularly advantageous for women, who often balance caregiving responsibilities, and working professionals who may struggle to find time for in-person appointments. By offering more flexible scheduling options, telemedicine improves the overall healthcare experience for individuals with demanding personal and professional schedules [33].

*Safety During the Pandemic:* The ability to access healthcare remotely became crucial during the COVID-19 pandemic, as it reduced the risk of viral transmission for both patients and healthcare providers. Telemedicine provided a safe alternative to in-person visits, allowing individuals to receive care without exposing themselves to crowded healthcare facilities. This aspect of telemedicine was particularly valuable during times of widespread lockdowns and restrictions, leading to its increased adoption across various patient groups during the pandemic [34].

*Better Chronic Disease Management:* Telemedicine has been especially beneficial for managing chronic diseases such as hypertension and diabetes. By enabling regular monitoring and timely healthcare interventions, telemedicine helps patients maintain better control of their conditions without the need for frequent hospital visits. This leads to improved patient outcomes, reduced hospital readmissions, and greater satisfaction among patients with chronic health conditions. The convenience and continuous care provided through telemedicine have shown significant positive impacts on the long-term management of chronic diseases [12].

### 5.1. Limitations of Telemedicine

Despite its advantages, telemedicine has several limitations that impact its effectiveness and equity; these are as follows. *Limited Physical Examination Capability*: Some clinical conditions require hands-on diagnostic assessments, which telemedicine cannot provide effectively. *Technological Barriers*: Poor internet connectivity, outdated digital devices, and limited digital literacy, especially in rural or underserved areas, continue to restrict access and reduce care quality. *Privacy Concerns*: Data security and confidentiality remain critical concerns for many patients using virtual care. *Reduced Personal Interaction*: The lack of face-to-face engagement can limit rapport-building between patients and providers, affecting trust and satisfaction. *Technology Dependence*: The success of telemedicine is closely tied to robust technology infrastructure, which is not uniformly available.

Addressing these challenges requires a multi-faceted approach focused on equity, infrastructure, training, and policy.

### 5.2. Strategies to Enhance Telemedicine Adoption

There are strategies to help enhance telemedicine adoption, which include the following: *Improving Digital Infrastructure in Rural Areas*: One of the major barriers to telemedicine adoption in non-metropolitan areas is poor digital infrastructure, including unreliable internet connections. Investment in broadband infrastructure is critical to ensuring that rural populations can access telemedicine services. This could involve government initiatives and public–private partnerships to enhance internet access, particularly in underserved regions, allowing patients to benefit from the convenience and accessibility of telemedicine. For example, the U.S. government’s Rural Digital Opportunity Fund (RDOF) is a multi-billion-dollar initiative aimed at expanding broadband access to rural communities, directly addressing the infrastructure gap that hinders telemedicine adoption [35].

*Increasing Technological Literacy*: Technological literacy, particularly among older adults and individuals in rural areas, plays a significant role in telemedicine adoption. Educational programs focused on teaching patients how to use telemedicine platforms, smartphones, and computers for virtual care can reduce the technology gap. Healthcare providers can offer instructional materials or workshops to help patients feel more comfortable with using telemedicine, thus encouraging wider adoption. For instance, local clinics in Kentucky initiated community outreach programs where nurses provided one-on-one training to elderly patients on how to use telemedicine apps, leading to a noticeable increase in adoption rates [36].

*Expanding Telemedicine Training for Healthcare Providers*: During the pandemic, healthcare systems were often overwhelmed by the rapid shift to telemedicine. Ensuring that healthcare providers receive adequate training in telemedicine practices can enhance the quality of care delivered via virtual platforms. Providers trained to deliver personalized care through telemedicine will likely improve patient satisfaction, addressing the concerns that may have led to a decrease in satisfaction among certain populations during the pandemic. For example, the American Medical Association (AMA) offers continuing education courses for healthcare providers on best practices for delivering care through telemedicine [1].

*Offering Telemedicine Services Tailored to Chronic Disease Management*: The data reveal that patients with chronic conditions like hypertension and diabetes showed high levels of satisfaction with telemedicine. Healthcare systems should prioritize expanding telemedicine programs focused on chronic disease management, providing regular check-ins and remote monitoring for these patients. This could reduce the burden of in-person visits while ensuring continuous care and improved health outcomes for patients managing long-term conditions. A program in California, called “Project ECHO”, has been successful in using telemedicine to manage chronic diseases by training healthcare providers in rural areas to offer specialized care to their patients via telehealth [37].

*Addressing Racial Disparities in Telemedicine Access*: The analysis showed that Black non-Hispanic and Hispanic populations experienced improvements in telemedicine access during the pandemic. However, disparities remain, and targeted outreach programs are needed to further increase telemedicine adoption in these communities. This could include language support, culturally appropriate healthcare services, and targeted efforts to raise awareness of telemedicine options within minority communities. For example, a telehealth initiative in South Texas addressed barriers for Hispanic populations by hiring bilingual staff and offering culturally tailored virtual consultations, which led to an increase in telemedicine usage [35].

*Developing User-Friendly Telemedicine Platforms*: Simplifying the user interface of telemedicine platforms is crucial for increasing adoption across diverse populations. Platforms should be designed with accessibility in mind, ensuring that patients of all ages and technological skill levels can navigate them easily. User-friendly features such as one-click access to appointments, clear instructions, and integration with wearable devices for remote health monitoring can improve the telemedicine experience and increase patient satisfaction. Telemedicine platforms like “AmWell” have successfully adopted such features, offering easy access to consultations, with integrations for remote monitoring devices to track vital signs in real time [37]. Emerging global telehealth platforms are becoming increasingly popular as consumers opt for digital health services due to lower costs, quicker responses from healthcare providers, and the added convenience of receiving care at home. However, integrating healthcare services across fragmented providers remains a challenge for telehealth platforms, making it difficult to offer seamless, one-stop solutions to patients without complex arrangements.

*Ensuring Affordability and Insurance Coverage*: Financial barriers can prevent patients from using telemedicine services. Ensuring that telemedicine is covered by insurance plans at rates comparable to in-person visits is critical for widespread adoption. Policymakers and healthcare providers need to work together to ensure that telemedicine services are affordable and accessible to all patients, regardless of their financial situation. For instance, during the COVID-19 pandemic, many insurance companies, including Medicare, expanded telehealth coverage, offering full reimbursement for virtual visits. This policy shift contributed to the rapid adoption of telemedicine across the United States [11].

## 6. Conclusions

This study offers a novel contribution by integrating causal loop modeling with survey-based quantitative analysis to examine factors influencing patient satisfaction with telemedicine before and during the COVID-19 pandemic. The findings suggest that telemedicine played a critical role in improving access to care, particularly among individuals with chronic conditions such as hypertension and diabetes, and in mitigating service delivery disruptions during the public health emergency.

Despite its contributions, the study is subject to certain limitations. The relatively modest sample size and reliance on descriptive statistics and one-sample *t*-tests necessitated by data constraints limit the depth of subgroup comparisons and the ability to control for confounding variables. Nevertheless, the emerging trends provide valuable preliminary insights that can inform healthcare policy and telemedicine implementation strategies.

To ensure the sustained and equitable adoption of telemedicine, targeted interventions are essential. These should include expanding digital infrastructure in underserved and rural areas, enhancing digital literacy across diverse populations, and addressing disparities in access among racial and ethnic groups. Furthermore, culturally responsive services and user-friendly telemedicine platforms will be vital to improving engagement and satisfaction across demographic lines.

While statistically significant differences were identified in several subgroup comparisons, the presence of wide confidence intervals and variability in means underscores the need for cautious interpretation. These inconsistencies may be attributable to small subgroup sizes or the influence of outliers. Future research should address these limitations by employing larger, more representative datasets and multivariate modeling techniques to validate and extend the current findings.

If implemented thoughtfully, telemedicine has the potential to advance a more accessible, efficient, and patient-centered model of healthcare delivery.

## Figures and Tables

**Table 1:** Inferential statistical analysis of patient responses.

Groups	Subgroups	Mean	95% CI (Lower)	95% CI (Upper)	*p*-Value
Age Group	18–44 years	434	157.73	710.94	0.010
	45–64 years	475	107.68	842.32	0.021
	65+ years	432	124.43	739.91	0.015
Sex	Male	606	271.80	941.87	0.006
	Female	775	218.99	1331.01	0.016
Urbanization	Metropolitan	1146	320.17	1971.83	0.016
	Non-Metropolitan	146	31.77	261.56	0.022
Race/Hispanic Origin	White NH	948	372.52	1523.48	0.008
	Black NH	325	93.29	556.71	0.015
	Hispanic	172	34.55	310.79	0.024
	Other Hispanic	220	4.92	436.75	0.047
Chronic Conditions	Hypertension	601	189.52	1012.48	0.013
	Diabetes	263	76.23	450.44	0.015
	Asthma	158	39.99	276.68	0.018

## Data Availability

The data utilized in this study were drawn from a nationally representative, publicly available survey administered by the CDC and provided by the National Center for Health Statistics, available at: https://data.cdc.gov/d/8xy9-ubqz (accessed on 18 June 2025).
